# Association between quality performance scorecard scores and health indicators: an ecological study in the Northern provinces of the Lao People’s Democratic Republic

**DOI:** 10.1186/s41182-025-00748-y

**Published:** 2025-05-21

**Authors:** Vixayyang Chayvangmanh, Noudéhouénou Credo Adelphe Ahissou, Khamsay Detleuxay, Daisuke Nonaka

**Affiliations:** 1 Department of Global Health, Graduate School of Health Sciences, University of the Ryukyus, 1076 Kiyuna, Ginowan, Okinawa 901-2720 Japan; 2 Department of Healthcare and Rehabilitation, Ministry of Health, Vientiane, Lao PDR

**Keywords:** Under-five mortality, Antenatal care, Outpatient services, Health outcomes, Laos, Healthcare quality

## Abstract

**Background:**

The Ministry of Health, Lao People's Democratic Republic (Lao PDR), surveyed health centers using the Quality Performance Scorecard (QPS) tool in 2021 to assess the quality of healthcare services at health centers. To validate the QPS tool, this study assessed the association between the QPS scores obtained from each health center and outpatient department (OPD) visits, antenatal care (ANC) coverages, and under-five mortality rates (U5MR) in the health centers' catchment area.

**Methods:**

This ecological study assessed the association between the QPS scores as an independent variable and OPD visits, coverage of ANC at least one visit (ANC1), coverage of ANC four or more visits (ANC4) and U5MR as a dependent variable, using secondary data collected from the 234 health centers and 31 district health offices in the four northern provinces, such as Huaphan, Xiengkhuang, Oudomxay and Phongsaly. Mixed-effect linear regression was used to assess the association between the independent variable and dependent variables while adjusting for covariates.

**Results:**

The mean value (standard deviation) was 64.9/100 (14.3) for QPS score, 3.3 (3.6) for U5MR (per 1,000 under-five population), 0.7 (0.5) for OPD visits (per population), 9.6 (6.0) for ANC1 coverage (per estimated number of reproductive-aged women) and 5.7 (4.4) for ANC4 coverage. The QPS scores were significantly independently associated with U5MR (unstandardized regression coefficient: −0.225 and standardized regression coefficient: −0.894), OPD visits (−0.004 and −0.114) and ANC4 coverage (−0.036 and −0.117).

**Conclusion:**

This study shows that with increasing level of quality of healthcare services as measured by QPS, the U5MR was decreasing. The association demonstrated the ability of the QPS tool to capture the quality of healthcare services at health centers. Although the QPS scores were also negatively associated with OPD visits and ANC4 coverage, these associations were weak and likely confounded by unmeasured factors, or explained by quality care potentially reducing patients’ perceived need for frequents visits. To further validate the QPS tool, a longitudinal study is recommended to confirm the findings and address unmeasured factors.

## Background

Ensuring high-quality healthcare is a global challenge, particularly in low- and middle-income countries. Suboptimal healthcare services contributes to poor health outcomes, accounting for an estimated 5.7–8.4 million deaths annually in low- and middle-income countries [1–3]. Even where healthcare services are available, inadequate infrastructure, substandard and medical products, and poorly trained providers often compromise their effectiveness [4–6].

Lao People's Democratic Republic (Lao PDR), a lower-middle-income country in Southeast Asia, delivers healthcare services through a decentralized system aimed at ensuring access to services across the country. The Lao public healthcare system includes five central hospitals, three specialist hospitals, 17 provincial hospitals, 135 district hospitals, and 1237 health centers [7]. Health centers in particular, provide approximately half of all healthcare services nationally: 62% family planning services for new users, 47% first antenatal care, and 40% outpatient services [8].

Meanwhile, the quality of healthcare services in Lao PDR remains challenging, with issues such as poor operational protocols, lack of essential equipment, inadequate infrastructure, workforce shortages, and low provider competencies, which affect healthcare services [9]. The poor quality of healthcare services might contribute to the high under-five mortality rate (U5MR) in the country (43 deaths per 1,000 live births in 2021), with substantial disparities between urban and rural regions (24 vs. 53) [10, 11]. A study conducted in malaria endemic districts of the country found the poor medication instruction of healthcare workers for malaria patients, which would be associated with poor medication adherence of patients [12]. Similarly, limited healthcare personnel and low-quality care at health centers and district hospitals were linked to severe dengue fever cases [13].

The Ministry of Health, Lao PDR, introduced the Quality Performance Scorecard (QPS) tool in 2020, developed by the Department of Healthcare and Rehabilitation in collaboration with central authorities and development partners to assess the quality of healthcare services at health centers [14]. The tool comprises 504 questions and evaluates various aspects of healthcare delivery through direct observation, staff interviews, records reviews, and infrastructure assessment.

The QPS assesses healthcare quality across three dimensions, such as structural quality, process quality and outcome quality. Structural quality accounts for 23% of total score and measures health center readiness, focusing on the availability of infrastructure, essential equipment, medicines, and supplies. The process quality covers 67% of total score and examines program management, infection control, cleanliness, and clinical competencies of health workers. Finally, with 10% of the total score, the outcome quality evaluates client satisfaction with randomly selected clients who used healthcare services in the previous three months [14]. In 2021, The Ministry of Health implemented a survey using the QPS tool in the Northern provinces, covering all 234 health centers in the four provinces, such as Huaphan, Xiengkhuang, Oudomxay, and Phongsaly. The survey results were detailed elsewhere [14].

Since the Lao PDR has adopted the QPS tool for quality assessment in health centers, no attempts have been made to validate the tool on the assumption that better quality of healthcare services increases service use and then leads to better health outcomes. Therefore, to validate the QPS tool, the present study assessed the association between the QPS scores obtained from each health center and OPD visits, antenatal care (ANC) coverages, and under-five mortality rates (U5MR) in the health centers' catchment area.

## Methods

### Study design

This study used an ecological design conducted at the health center level to assess the association between QPS scores and key healthcare indicators such as ANC, OPD, and U5MR. The ecological design was used because the QPS tool assessed the healthcare characteristics at the health center level.

### Study site

The study used secondary data from 234 health centers across the four Northern provinces, based on the availability of QPS data: 75 health centers in Huaphan, 56 in Xiengkhuang, 53 in Oudomxay, and 50 in Phongsaly. The Ministry of Health and World Bank initially selected these four provinces due to their highest concentration of various ethnic groups.

### Data collection

We obtained data on QPS from the Department of Healthcare and Rehabilitation, Ministry of Health; and data on U5MR, OPD, and ANC use from the 31 district health offices in the four provinces. Additionally, we obtained data on staff numbers, health center locations, and population from the district health offices.

### Variables

#### Independent variables

The main independent variable was the total score of QPS, which ranged from 0 to 100 and consisted of three component variables: structural quality score, process quality score, and outcome quality score. The structural quality dimension score (from 0 to 23) was generated from the responses to the 33 weighted questions, and the process quality score (from 0 to 73) was generated from the responses to the 223 weighted questions. Finally, the outcome quality score ranges from 0 to 10 and was generated from the responses to the 22 weighted questions.

#### Dependent variables

This study has four dependent variables: U5MR, ANC coverage of at least one visit (ANC1 coverage), ANC coverage of at least four visits (ANC4 coverage), and OPD visits. Ideally, U5MR should be calculated as the number of under-five deaths per 1,000 live births. However, the present study used the under-five population as the denominator since the accurate number of live births could not be confirmed due to prevalent home-based childbirths. Therefore, the U5MR was calculated as the number of under-five deaths per 1000 under-five population recorded in 2021 within each health center's catchment area. Given that many areas reported no under-five deaths in some years, we used a 3-year average (2021, 2022, and 2023).

Similarly, ANC coverage is commonly calculated using the number of pregnant women as the denominator. However, such data not being available, we used an estimated number of women of reproductive age (16–49 years old) as a proxy. Thus, the ANC1/ANC4 coverage was calculated as the number of pregnant women who visited the health center at least once/four times in 2021 divided by the estimated number of reproductive-aged women. We estimated the number of reproductive-aged women on the assumption that reproductive-aged women constitute approximately 56% of the total female population, according to the population distribution by age groups reported in the 2021 Statistical Yearbook [7]. The 2022 Statistical Yearbook also reported a similar percentage (54%) [15]. Lastly, the OPD visits was calculated as the number of outpatient visits recorded in 2021 divided by the population in the same year.

### Covariates

The study included four covariates: staff-to-population ratio, health center type, distance from the health center and the district hospital, and the health center catchment population. First, the staff-to-population ratio was calculated as the number of healthcare workers at each health center divided by the population recorded in 2021 in the health center's catchment area. This variable was then categorized into quartiles for multivariate analyses. Second, the health center type was a variable with two categories: Type A health centers, which provide child delivery services, and Type B health centers, which do not offer child delivery services but provide prenatal and postnatal care. Third, the distance between the health center and the district hospital was the road distance expressed in kilometers (km). This variable was also categorized into quartiles for multivariate analyses. Finally, the health center's catchment population was the total population recorded in 2021 in the health center's catchment area, which was similarly categorized into quartiles for multivariate analyses.

### Statistical analysis

First, descriptive analyses were conducted to summarize continuous variables using means with standard deviations and medians with interquartile ranges, while categorical variables were summarized using frequencies and proportions. Second, using Spearman's correlation test, we performed bivariate analyses to examine the linear relationship between independent and dependent variables. Third, multivariate analyses were conducted to assess the association between the main independent variable and dependent variables while adjusting for covariates. Linear regression was used for the multivariate analyses, and multi-level modeling was applied to account for the hierarchical structure of the data, where health centers were nested within districts and districts were nested within provinces. All the analyses were performed using IBM SPSS Version 26.

## Results

### General characteristics of the health centers

Most health centers (85.5%, *n* = 200) were Type B facilities, where child delivery services were unavailable (Table 1). The median number of staff per health center was 4 (interquartile range: 3–5). Qualified staff were available in most health centers: midwives (89.7%), medical doctors (86.3%), and nurses (84.6%). Each health center was responsible for a median of 6 villages (interquartile range: 4–8), covering a median population of 2837.

**Table 1:** Characteristics of study health centers (*n* = 234)

Characteristics	*n* (*n* = 234)	%
Health center type		
A (with child delivery services)	34	14.5
B (without child delivery services)	200	85.5
Staff number		
Median (interquartile range)	4	(3–5)
Staff-to-population ratio		
Median (interquartile range)	14.3	(9.9–18.9)
Health center with the following qualified staff		
Midwife	210	89.7
Medical doctor	202	86.3
Nurse	198	84.6
Laboratory technician	7	3.0
Other	54	23.1
Distance between health center and district hospital; km		
Median (interquartile range)	31	(21–54)
Number of responsible villages		
Median (interquartile range)	6	(4–8)
Population in the responsible villages		
Median (interquartile range)	2837	(1988–3686)
Number of under-five children in the responsible villages		
Median (interquartile range)	320	(216–420)

### Quality performance scorecard (QPS)

The mean score (standard deviation) for the total QPS was 64.9 (14.3), with 13.5 (5.3) for the structural quality dimension, 45.7 (10.3) for the process quality dimension, and 5.7 (1.8) for the outcome quality dimension (Table 2).

**Table 2:** Scores of Quality Performance Scorecard (QPS)

Dimension	Mean	Standard deviation	Median	Interquartile range
Structural quality	13.5	5.3	11.0	8.0–19.0
Processes quality	45.7	10.3	45.9	35.5–54.0
Outcome quality	5.7	1.8	6.0	5.0–7.0
Total	64.9	14.3	64	52.6–76.9

### Under-five mortality rate (U5MR), OPD visits, and antenatal care (ANC) coverage

The mean value (standard deviation) was 3.3 (3.6) for U5MR, 0.7 (0.5) for OPD visits, 9.6 (6.0) for ANC1 coverage, and 5.7 (4.4) for ANC4 coverage (Table 3).

**Table 3:** Under-five mortality, antenatal care coverage and outpatient department use

Dependent variables	Mean	Standard deviation	Median	Interquartile range
Under-five mortality rate (number of under-five deaths per 1000 under-five children per year)	3.3	3.6	2.3	1.1–4.5
Outpatient department service visits (number of outpatient-department use per population per year)	0.7	0.5	0.6	0.3–1.0
Antenatal care coverage of at least one visit (number of pregnant women making at least one antenatal care visit per estimated number of reproductive-aged women)	9.6	6.0	8.7	6.2–12.0
Antenatal care coverage of ≥ four visits (number of pregnant women making four or more antenatal care visits per estimated number of reproductive-aged women)	5.7	4.4	5.0	3.0–7.1

### Spearman's correlation between the independent and dependent variables

Overall, the total QPS score showed a significant inverse correlation with all the dependent variables (Table 4). Regarding the individual component of the total QPS score, the structural quality dimension score had a significant negative correlation with OPD visits only, while the process quality dimension score also had a significant negative correlation with all the dependent variables. Lastly, the outcome quality dimension score had a significant negative correlation with U5MR only.

**Table 4:** Correlation between the scores of Quality Performance Scorecard and under-five mortality rate, outpatient department use and antenatal care coverage (Spearman’s correlation test)

	Under-five mortality rate		Outpatient department service use		Antenatal care coverage of ≥ one visit		Antenatal care coverage of ≥ four visits	
Quality performance scorecard	Correlation coefficient	*p*-value	Correlation coefficient	*p*-value	Correlation coefficient	*p*-value	Correlation coefficient	*p*-value
Structural quality	− 0.054	0.415	− 0.449	< 0.001	− 0.091	0.165	− 0.100	0.126
Process quality	− 0.383	< 0.001	− 0.325	< 0.001	− 0.150	0.022	− 0.185	0.005
Outcome quality	− 0.182	< 0.001	− 0.052	0.425	0.031	0.637	0.029	0.655
Total	− 0.318	< 0.001	− 0.405	< 0.001	− 0.137	0.036	− 0.166	0.011

### Results of regression analyses

In the bivariate analyses where the covariates were not included in the model, the total QPS score was significantly associated with U5MR (unstandardized regression coefficient: − 0.215), OPD visits (− 0.005), ANC1 coverage (− 0.058), and ANC4 coverage (− 0.053) (Table 5). Meanwhile, in the multivariate analyses, after adjusting for covariates, the total QPS score remained significantly and independently associated with U5MR (unstandardized regression coefficient: − 0.225), OPD visits (− 0.004), and ANC4 coverage − 0.036).

**Table 5:** Association between the total scores of Quality Performance Scorecard and under-five mortality rate, outpatient department use and antenatal care coverage

	Bivariate analyses (unadjusted results)				Multivariate analyses (adjusted results)^α^					
	Unstandardized				Unstandardized		Standardized			
Dependent variables	Regression coefficient	SE^β^	*t*	*p*-value	Regression coefficient	SE^β^	Regression coefficient	SE^β^	*t*	*p*-value
Under-five mortality rate	− 0.215	0.026	− 8.034	< 0.001	− 0.225	0.024	− 0.894	0.095	− 9.296	< 0.001
Outpatient department visits	− 0.005	0.002	− 2.541	0.012	− 0.004	0.009	− 0.114	0.257	− 4.864	< 0.001
Antenatal care coverage of ≥ one visit	− 0.058	0.016	− 3.603	< 0.001	− 0.036	0.022	− 0.086	0.052	− 1.638	0.103
Antenatal care coverage of ≥ four visits	− 0.053	0.010	− 4.921	< 0.001	− 0.036	0.011	− 0.117	0.036	− 3.165	< 0.001

^α^Adjusted for staff-to-population ratio, health center type, distance from health center to district hospital and health center catchment population

^β^Standard error

## Discussion

Our analysis showed that the total QPS scores were significantly inversely associated with U5MR, suggesting that better quality of healthcare services provided by health centers led to lower under-five mortality rates in the catchment area of health centers. The observed association is likely to reflect a causal relationship between the variables for two key reasons: first, the effect size measured by the standardized regression coefficient was large (i.e., − 0.894), indicating a strong association. Second, the association is plausible [16, 17]: World Health Organization reports that 60% of deaths in low- and middle-income countries from conditions requiring healthcare occur due to poor-quality healthcare [2]. In addition, among the three dimensions of healthcare quality examined in the present study, the process quality dimension had the strongest correlation with U5MR (correlation coefficient: 0.383). This dimension assessed clinical skills, outreach services, infection control, and hygiene, which are linked to child survival. Similar studies conducted in Vietnam and sub-Saharan Africa have demonstrated that among various healthcare quality domains, provider competency and diagnostic capacity, which are the key aspects of process quality in our study, were most strongly associated with improved service readiness [18, 19]. Furthermore, a large cross-sectional study analyzing 3,390 child deaths in sub-Saharan Africa and Southern Asia found that 77% of them could be prevented by improved clinical management and quality care [20].

Our study also found a significant negative association between total QPS scores and OPD visits. However, such an inverse association has not been previously reported and, a priori, is less likely to reflect a causal relationship. First, it is generally accepted that low-quality healthcare services tend to result in lower rates of OPD visits [21]. For example, a qualitative study in Vietnam reported that when primary care facilities were perceived to offer lower-quality services, patients often bypassed them in favor of higher-level facilities [22]. Second, the effect size observed in our study, as indicated by the standardized regression coefficient (− 0.114), was small, suggesting a weak association between the two variables [16, 17]. Therefore, this relationship may be confounded by unmeasured factors including geographical accessibility to healthcare facilities a known determinant of OPD visits [21, 23, 24]. Although our analysis adjusted for the distance between health centers and their respective referral district hospitals, village-level factors could not be adjusted for as the unit of analysis was the health center.

Alternatively, one possible explanation for the negative association between QPS scores and OPD visits is that patients who receive high-quality of healthcare services may perceive less need for repeated OPD visits. Several studies have highlighted how key components of quality of healthcare services can influence the perceived need for healthcare services, potentially reducing frequent demands [25–27]. In our analysis, the inverse association between overall quality of healthcare services and OPD visits appeared to be largely driven by structural quality (23% of the total QPS score;* r* = − 0.449, *p* < 0.001) and process quality (67% of the total score;* r* = − 0.325, *p* < 0.001), while outcome quality (10% of the total score) showed no significant correlation. It is likely that health centers with better infrastructure, skilled staff, and efficient service delivery help reduce the perceived need for unnecessary OPD visits by effectively managing minor illnesses early and promoting preventive care [25].

Similarly, a significant negative association was found between total QPS scores and ANC4 coverage. As with OPD visits, this association was unexpected and likely not causal, given the small effect size (standardized coefficient = − 0.117) and the fact that, in theory, better quality of healthcare services should attract more pregnant women [16, 17]. Several contextual factors may explain this finding. First, the COVID-19 pandemic between 2019 and 2022 might have negatively impacted pregnant women's ANC visits, as pregnant women were particularly vulnerable during the pandemic [8, 28]. Pregnant women might avoid visiting health centers with better quality of healthcare services, as such centers are likely to be crowded. Second, village health volunteers, who play a key role in promoting ANC visits, may have had a greater influence in certain catchment areas, particularly those with lower-quality services [29, 30].

Meanwhile, one possible explanation for this inverse association relates to the role of process quality, which reflects providers’ clinical competencies and adherence to guidelines. In our analysis, the negative correlation between total QPS scores and ANC4 coverage (*r* = − 0.166, *p* = 0.011) was primarily driven by this component. Just as comprehensive care in high-quality health centers may reduce the perceived need for subsequent OPD visits, health centers with well-trained staff may offer more comprehensive ANC during each visit, reducing the perceived need for multiple follow-ups [25]. In high-quality health centers, fewer visits may not indicate reduced utilization but may highlight rather more potential efficient care, with improved counseling, timely screenings, and early management of pregnancy risks in those settings. The non-significance of the association between the overall QPS score and ANC1 emphasizes this possibility.

## Limitations

The present study has three major limitations. First, we used the estimated number of reproductive-aged women to calculate the ANC coverages instead of the actual number of pregnant women. This approach may lead to an overestimation of ANC coverage in some health center catchment areas and an underestimation in others, as we used the same factor (i.e., 0.56) across all the health centers to estimate the number of reproductive-aged women from the total female population. Because of the limitation, our analysis assessing the association with ANC coverages would not be sensitive very much. Second, instead of the number of live births, we used the number of under-five children as the denominator to calculate the U5MR. Consequently, the U5MR reported in this study is not directly comparable to the general U5MR. Third, in our analysis we did not include economic and educational characteristic because such characteristic data were not available at health center level. Nevertheless, the impact of the omission is likely minimal as the health center zones share similar economic characteristics and educational background.

## Conclusion

This study shows that QPS scores were negatively and strongly associated with U5MR in the health center catchment areas. On the assumption that better quality of healthcare services leads to better health outcomes, including lower U5MR, the association demonstrated QPS's ability to capture the quality of healthcare services at health centers. Although the QPS scores were also negatively associated with OPD visits and ANC4 coverage, these associations were weak and not plausible. Nonetheless, these associations may highlight a complex process through which quality components influence the perceived need for repeated demand for care. These associations are likely to be confounded by unmeasured factors known to influence healthcare service utilization (e.g., geographical accessibility from villages to health centers). To further validate the QPS tool, a retrospective cohort study is recommended that helps to confirm the findings while addressing the limitations of the present study.

## Data Availability

The data generated and analyzed in the current study are available from the corresponding author upon a reasonable request.
